# Use of post-graduate students' research in evidence informed health policies: a case study of Makerere University College of Health Sciences, Uganda

**DOI:** 10.1186/s12961-018-0343-8

**Published:** 2018-08-03

**Authors:** E. A. Obuku, N. K. Sewankambo, D. K. Mafigiri, F. Sengooba, C. Karamagi, J. N. Lavis

**Affiliations:** 1 Clinical Epidemiology Unit, Department of Medicine, School of Medicine, College of Health Sciences, Makerere University, P.O. Box 7072, Kampala, Uganda; 2 Department of Health Research Methods, Evidence and Impact, McMaster University, Hamilton, Canada; 3 Department of Health Policy and Planning, School of Public Health, College of Health Sciences, Makerere University, Kampala, Uganda; 4 Department of Social Work and Social Administration, College of Humanities and Social Sciences, Makerere University, Kampala, Uganda; 5 Regional East African Community Health (REACH) Policy Initiative, College of Health Sciences, Makerere University, Kampala, Uganda; 6 Department of Global Health and Population, Harvard School of Public Health, Harvard University, Massachusetts, United States of America; 7 Center for Social Science Research on AIDS, Department of Anthropology, College of Arts and Sciences, Case Western Reserve University, Cleveland, OH United States of America; 8 Faculty of Epidemiology and Population Health, London School of Hygiene and Tropical Medicine, London, United Kingdom

**Keywords:** Student, Post-graduate, Research, Evidence-informed health policy, Uganda

## Abstract

**Background:**

World over, stakeholders are increasingly concerned about making research useful in public policy-making. However, there are hardly any reports linking production of research by students at institutions of higher learning to its application in society. We assessed whether and how post-graduate students’ research was used in evidence-informed health policies.

**Methods:**

This is a multiple case study of master’s students’ dissertations at Makerere University College of Health Sciences (MakCHS) produced between 1996 and 2010. In a structured review, we applied a theoretical framework of ‘research use’ and used content analysis to map how research was used in public policy documents. We categorised content of these documents according to the health-related Millennium Development Goals (MDG). We defined a case of ‘use’ as citation of research products from a master’s student’s dissertation in a public policy-related document.

**Results:**

We found 22 cases of research use in policy-related documents (0.5%) out of a total 4230 citations from 16 of 1172 total dissertations (1.4%). Additionally, research was mostly cited in primary studies (95.4%), systematic reviews (3%), narrative reviews (0.8%) and cost-effectiveness analyses (0.2%). Research was predominantly used instrumentally, to either frame the problem (burden of disease or health condition) or select an intervention (treatment or diagnostic option) and rarely symbolically to justify strategies already selected. The bulk of the cases of research use addressed child health (MDG 4), focusing on infectious diseases (MDG 6), mainly in international clinical or public health guidelines, working papers, a consensus statement and a global report. We distilled ‘synergistic relationships’ among organisations or interest groups, ‘globalisation of local evidence’, ‘trade-offs’ in the use of research and use of ‘negative results’ from the documents and text content.

**Conclusions:**

Research from dissertations of post-graduate students at MakCHS is used in evidence-informed health policies, particularly for infectious diseases in child health. Further, we have delineated pathways of research use in the global arena and highlighted the importance of ‘negative results’ from dissertations of post-graduate students at MakCHS.

## Background

World over, stakeholders are increasingly concerned about making research useful in public health policy-making [1]. In fact, to paraphrase the title of one article, the paradox of health services research; if it is not used, why produce so much of it? [2, 3]. Since Archie Cochrane’s seminal work on ‘effectiveness and efficiency’ in the delivery of health services in 1972 [4], the evidence-based medicine movement has evolved to other spheres of public health policy [5]. Indeed, high-level meetings of researchers and government actors in Mexico in 2004, Bamako in 2008 [6] and regional knowledge translation platforms including in East Africa [7] have been instrumental in propagating this momentum of evidence-informed health policy (EIHP). More recently, the 2013 World Health Report called for evidence to drive the scale-up of universal health coverage [8]. It is therefore incumbent upon institutions of higher learning to produce research that is relevant to bridge the information gaps that will advance social services in society, including better-informed healthcare [9, 10].

While studies on publication of students’ research are prevalent, including in systematic reviews [11–14], there are hardly any reports linking production of research by post-graduate students at institutions of higher learning to its application in public policy. Churning out research for academic incentives such as publication and career advancement is necessary but not sufficient in the spectrum of utility of research findings. Previous work on research use in decision-making has focused on barriers, facilitators, tools and interventions in general terms [3, 15–17]. On the one hand, evidence from systematic reviews documented perceived facilitators to the use of research in decision-making as personal contact and skills-building with policy-makers, timely relevance, and the inclusion of summaries with policy recommendations. On the other hand, perceived irrelevance of the research, negative attitude towards use of research evidence, lack of appropriate skills by decision-makers to find, read, appraise and interpret research evidence, use of jargon in research evidence brief write ups, mutual mistrust, and power and budget struggles were documented as barriers [17–22].

Noteworthy, specific attention has not been given to the unique enclave of graduate students’ research and its use. Post-graduate students, unlike faculty researchers, for example, subsist in a training environment of which writing a thesis is a fundamental step. Generally, the publication of post-graduate students’ research follows the same processes as other research. After completion of the thesis, a manuscript would be written and submitted to an appropriate journal. Although this process inculcates knowledge and skills for scientific enquiry, critical thinking and systematic problem-solving, post-graduate students may not have the incentives to advance their research projects beyond theses as a requirement to graduate. For example, a study at Johns Hopkins School of Public Health, United States of America found that only 47% of students’ research compared to 73% of other investigators got published [23]. It is against this background that we assessed the utility of post-graduate students’ research in EIHP at Makerere University in Uganda, a low-income country.

Makerere University College of Health Sciences (MakCHS) is a pioneer health professions education and research institution in Africa, established in 1924 [24]. In the recent past, Makerere University has been highly ranked in Africa in terms of research productivity [25]. Post-graduate students at MakCHS are required to conduct a research project before completion of their degree course. During this process, at least one academic supervisor (faculty) supports the student to produce a dissertation over a 2- to 4-year period of study. Publication and translating the research findings has not been a requirement for graduation. The publication of post-graduate students’ research follows the same processes as other research, namely the writing and submission of a manuscript to an appropriate journal following completion of the thesis.

## Methods

### Theoretical underpinnings

We based our study on the theoretical concepts of ‘research use’ posited by Carol H. Weiss in 1979 [26]. Weiss describes seven models of research use, broadly grouped as linear (‘knowledge driven’ or ‘problem solving’) or complex (‘interactive’, ‘enlightenment’, ‘political’, tactical or ‘intellectual enterprise’). The ‘knowledge-driven model’ assumes that the mere existence of knowledge discloses opportunity for public policy relevance. In the ‘problem-solving model’, a pending decision is caused by gaps in knowledge which, when filled by new information from research, a solution is provided.

We do not apply the complex models of Weiss and hence do not describe the details here. Generally, the complex models entail the play of multiple factors that cause diffusion of knowledge into the decision-making process. The use of research is an input of a complicated process that includes experience, political insight, pressure, social technologies and judgment in the interactive model, while the tactical model entails decision-making bodies referencing research to delay a decision or deflect criticism. In the political model, interests, intellect and ideology pre-define the positions of the decision-makers that research is unlikely to shake. Enlightenment is said to occur when findings of a single study or a body of related studies do not directly affect policy, and instead concepts and theoretical perspectives permeate the policy-making process. In essence, political and enlightenment are the same as political and conceptual use. Finally, research is viewed as an intellectual enterprise of society and not an independent variable affecting policy, so much so that research is influenced by the larger fashions of societal thought.

We use the three concepts as applied in the work of Lavis et al. [3], who merged the Weiss model of research use into instrumental, conceptual or symbolic. A decision-maker acting on research findings directly and specifically to solve a problem at hand makes ‘instrumental use’ of research (problem-solving model). In this instrumental model, research is often used in a very direct way, such as to change protocols. In ‘conceptual use’, research is applied generally and indirectly to enlighten the ‘informed publics’ to the extent that, without any special effort, ‘truth will triumph’. As such, in conceptual use of research, the actor rarely pinpoints citable research material and instead relies on incremental exposure to ideas and orientations from numerous research findings. In conceptual use, research is applied indirectly and changes the way that individuals think about a problem, options or implementation considerations. Further, in practice, conceptual use is thought to happen more frequently than instrumental use, although in a less tangible way. ‘Symbolic use’ of research depicts ‘after the fact’ application of research results, commonly to justify a decision already taken. This is observed where indecision on a public issue has been prolonged and opinions have hardened, without room for entertaining new evidence by actors. In other words, symbolic use is persuasive application of research as a political tool to influence or legitimate policies and decisions made for other reasons.

### Design

In light of the theoretical frameworks above, we conducted a multiple case study [27] of 22 citations from 16 research dissertations of post-graduate students at MakCHS who matriculated between 1996 and 2010. We adapted approaches used by previous scholars of research to policy [3], that is, identifying a sample of policy-related documents by tracking citations of products of the post-graduate students dissertations. We followed this by assessing the content of the text that cited the research and, where possible, mapped contextual issues around the use of research. We broadly characterised a document as ‘policy related’ if it highlighted a key health issue or provided direction to the field in the form of working papers, consensus statements or programme reports. We excluded editorials, opinion pieces or books. We categorised the policy-related documents by Millennium Development Goals (MDGs), which was the globally agreed priority benchmark for measuring social progress from 2000 to 2015 [28].

### Data collection

We reviewed all 1172 dissertations available at the archive of post-graduate dissertations of MakCHS. All the dissertations were from the period 1996 to 2010. Using a paper-based tool, we manually extracted data from hard copies of research dissertation reports and validated these using the Makerere University institutional online repository of research theses [29]. We searched Google scholar (https://scholar.google.com/) and PubMed (https://www.ncbi.nlm.nih.gov/pubmed/) to identify policy-related documents citing products of dissertations of the post-graduate students up to June 2016. We found a total of 196 dissertations that were cited, out of the 1172 dissertations, of which 16 were cited by policy-related documents and the rest in peer-reviewed manuscripts.

We captured data from the citing document about the post-graduate student dissertation that was cited (author, year, title, research design) and about the citing document (type of document, owner of the document, the phrase citing work from the post-graduate student dissertation).

### Coding and synthesis of the data

We coded for phrases or sentences where the research was being cited in the policy-related documents. We defined a ‘case of research use’ as citation of research products from post-graduate students’ dissertations in publicly available health policy-related documents [3, 30]. We interpreted meaning from the text data using directed content analysis [31], in which a theory or relevant research findings provide guidance for the initial codes. We assessed whether the research was cited instrumentally such as to frame a health problem (burden of disease or health condition) or select an intervention (treatment or diagnostic option), or symbolically to justify strategies already selected. We broadened our assessment of symbolic use to include contextual issues surrounding the health problem under consideration and framing of the language text in applying the research. We created new codes for any text or observation that could not be categorised with the initial coding scheme to explain unique phenomenon [31]. The doctoral student (EAO) developed the codes, which were corroborated by a research associate (MO). We resolved any differences in the coding by discussion.

### Ethical considerations and approvals

The School of Medicine Research & Ethics Committee, the Uganda National Council for Science & Technology (HS 3268) and Office of the President of Uganda (ADM/154/212/01) approved this study. Since we used data that was available in the public domain, we characterised this study as having less than ‘minimal risk’ [32].

## Results

### Documents citing research products of post-graduate students at MakCHS

Overall, there were 4230 citations from products (dissertations and peer-reviewed articles) out of 1172 total post-graduate research projects at MakCHS in the period under review. Most (95.4%) of these citations were by other primary studies, while 4.6% were citations by evidence syntheses. These evidence syntheses included systematic reviews (130, 3%), non-systematic reviews (35, 0.7%) and cost-effectiveness analyses (6, 0.2%). There were 196 (17%), 85 (7.2%) and 16 (1.4%) unique dissertations cited by peer-reviewed primary research work, evidence syntheses and policy-related documents, respectively (Table 1). None of the dissertations were evidence syntheses.

**Table 1:** Types of documents citing products of post-graduate students’ research dissertations at MakCHS 1996–2010

Type of document^a^	All citations *N* = 4230	Unique dissertations cited *N* = 1172
Primary research work	4,037 (95.4%)	196 (17%)
Evidence syntheses	193 (4.6%)	85 (7.2)
Policy related^b^	22 (0.5%)	16 (1.4%)
Systematic reviews	130 (3.0%)	67 (5.7)
Non-systematic reviews	35 (0.8%)	26 (2.2%)
Cost-effectiveness analyses	7 (0.2%)	5 (0.4%)

^a^Documents include peer and non-peer reviewed ones, published and grey literature

^b^Broadly characterised a document as ‘policy related’ if this document highlighted key health issue or provided direction to the field such as that guidelines, working papers, consensus statements or programme reports

**Table 2:** Description of research use and policy-related documents citing products of post-graduate students’ research dissertations at MakCHS 1996–2010

Characteristic	Citations *N* = 22
Type of research use	
Instrumental	20
Symbolic	2
Conceptual	0
Citing document	
Guideline – Clinical/Public health	13
Working paper (Advocacy)	7
Consensus statement	1
Global report	1
Citing organisation	
WHO	10
Network of Community of Practice	8
State department/Ministry	3
International NGO	1
Priority area cited	
MDG 4 (Child health)	14
MDG 5 (Maternal health)	1
MDG 6 (Malaria, HIV, TB)	17
Study design of cited research	
Cross-sectional	9
Randomised control trial	7
Cohort	5
Case control	1

We observed that it was possible for two or more research products to be cited in a single policy-related document. We report 19 distinct documents that cited research products of post-graduate students at MakCHS. The types of policy-related documents citing research products from dissertations of post-graduate students at MakCHS were predominantly international clinical or public health guidelines (64%), working papers (27%), a consensus statement (4.5%) and a global report (4.5%). These documents belonged mostly to networks of communities of practice made up of professional associations and academia (56%) such as medical professional associations in the United States, Britain and South Africa, WHO (46%), government departments (14%) and an international non-governmental organisations (Marie Stopes International) (4.5%) (Table 2).

### Description of utilisation of research from post-graduate students’ theses

We found 22 cases of research use (0.5%) in health policy-related documents, out of 4230 citations. These citations were from 16 of 1172 dissertations available for study (1.4%), with four dissertations being cited in two or more policy-related documents. Research was chiefly used instrumentally, to either frame the problem (burden of disease or health condition) or to choose or justify strategies to select an intervention (treatment option). In two instances, research was applied symbolically to reinforce a decision already taken (Table 3). We did not find an instance where research was applied conceptually.

**Table 3:** Description of text content of ‘how research was used’, from products of masters’ students’ dissertations

Student and thesis year	Research use	Description of text citing research product	Title of citing document
Aceng, 2003	Instrumental (treatment option)	When these children were analysed, “… *eleven other trials found no significant difference in mortality between artemisinin derivatives and quinine …the frequency of serious AEs was small in most studies and no statistical inferences can be drawn…*”	The WHO Pocketbook of Hospital Care for Children: [Evidence behind the WHO Guidelines: Hospital Care for Children: Efficacy and Safety of Artemisinin Derivatives in Children with Malaria] [58]
Achan, 2004	Instrumental (treatment option)	In a meta-analysis of comparison of the effect of intrarectal or intravenous administered quinine showed reduced mortality in African children, RR 0.77, 95% CI 0.22–2.72; this study contributed 42.7% of the data to the meta-analysis results, RR 0.55, 95% CI 0.24–1.24	Treatment of African children with severe malaria-towards evidence-informed clinical practice using GRADE [59]
Alia, 2002	Symbolic (tactical) (justify a decision)	A wealth of scientific evidence, *“…including numerous randomized and comparative clinical trials…supports our view that misoprostol should be included in the EML for this indication…for treatment of incomplete abortion and miscarriage…high success rates, in the range of 90 to 100%…*”	Support of Gynuity Health Projects’ application for misoprostol to be added to WHO’s Essential Medicines List (EML) for the indication of treatment of incomplete abortion and miscarriage [60]
Bongomin, 2003	Instrumental (frame a problem)	“*…Our study confirms the high prevalence of hepatitis B infection among health personnel in Uganda…consistent…with previous studies in…medical students at Mulago National Referral Hospital…11%, respectively, to be chronic carriers. Age and duration of service have similarly been associated with an increase in hepatitis B sero-positivity in other surveys in health personnel…infection continues in adulthood through sexual transmission…*”	Hepatitis B infection among health workers in Uganda: evidence of the need for health worker protection [61]
Iriso, 2002	Instrumental (diagnostic option)	“*…Diagnosis: A standardized protocol that includes...the potential utility of induced sputum…obtaining diagnostic specimens from children in the outpatient setting…*”	Guidelines for the prevention and treatment of opportunistic infections among HIV-exposed and HIV infected children: recommendations from CDC, the National Institutes of Health, the HIV Medicine Association of the Infectious Diseases Society of America, the Pediatric Infectious Diseases Society, and the American Academy of Pediatrics [62]
Kambugu, 2002	Instrumental (treatment option)	“*…Finally, several clinical trials, many undertaken in Southern Africa, have improved our understanding of issues such as…how to safely administer amphotericin B deoxycholate, which has been used by many more South African clinicians as first-line induction-phase treatment for CM in the last 5 years…*”	Guideline for the prevention, diagnosis and management of cryptococcal meningitis among HIV-infected persons: 2013 update [63]
	Symbolic (tactical) (justify a decision)	“*…We and others have previously demonstrated manageable and reversible renal impairment in cohorts of HIV-infected patients managed with AmBd at 0.7–1 mg/kg/day with careful pre-hydration and electrolyte monitoring and supplementation, without a requirement for renal replacement therapy…*”	British HIV Association opportunistic infection guidelines: in defense of amphotericin B deoxycholate [64]
Kitaka, 2002	Instrumental (frame a problem)	“*… Respiratory samples from pneumonia patients in clinical studies (%)… isolated from NPA and IS...Pneumocystis jiroveci* [found in] *18 of 43 HIV positive children aged 2–60 months (41.9%) and 2 of 78 HIV negative (2.6%)…*”	The WHO Pocketbook of Hospital Care for Children: [Evidence behind the WHO Guidelines: hospital care for children: what is the etiology of pneumonia in HIV-infected children in developing countries?] [34]
	Instrumental (diagnostic option)	“*…study of Ugandan children with severe pneumonia reported that those with PCP had a smaller head circumference compared to those without PCP (41.4 cm compared with 44.4 cm, p < 0.0001)…*” “*…Similarly, Bakeera–Kitaka, et al. found mean levels of 816 units/L in PCP compared with 568 units/L in others (p = 0.004). Those died from PCP, in this study, had significantly higher levels than survivors. This is supported by evidence from developed countries…*”	The WHO Pocketbook of Hospital Care for Children: [Evidence behind the WHO Guidelines: hospital care for children: what is the etiology of pneumonia in HIV-infected children in developing countries?] [34]
Mpimbaza, 2007	Instrumental (treatment option)	“*… Outcome 1: Seizure cessation within 10 minutes…allocation concealment only mentioned in Mpimbaza 2008…recommended doses is usually about 0.3–0.5 mg/kg in children…and the rest of the adverse events studies did not report any serious adverse effect with use of buccal midazolam…*”	Application for inclusion of buccal midazolam in the WHO List of Essential Medicines: (submitted to) Expert Committee on the Selection and Use of Essential Medicines [42]
	Instrumental (treatment option)	“*…the superior effectiveness and safety of buccal midazolam compared to rectal diazepam came from…three RCTs…although the evidence for effectiveness came from studies in both high-income and resource-poor countries, the majority of the evidence for efficacy and safety had been generated from its use in accident and emergency departments and not at community level…buccal midazolam is not available in many…*”	The selection and use of essential medicines: Report of the WHO Expert Committee, 2009 (including the 16th WHO Model List of Essential Medicines and the 2nd WHO Model List of Essential Medicines for Children), WHO Technical Report Series [65]
	Instrumental (treatment option)	“*…a greater amount of time is required to place the IV than to deliver* [midazolam] *by non-IV routes…therefore, the total amount of time from the decision to treat with a benzodiazepine to seizure cessation is equivalent or less when alternative routes are used…has been shown for midazolam when delivered by…buccal routes…*” “*…we recommend buccal midazolam over rectal diazepam for pre-hospital seizure cessation and control... low evidence quality and strong recommendation…randomized trials have suggested…buccal midazolam leads to more frequent seizure cessation than rectal diazepam…no difference in respiratory arrest or depression…*”	An evidence-based guideline for pediatric pre-hospital seizure management using grade methodology [66]
	Instrumental (treatment option)	“*...the largest study of 330 children in Uganda found a lower rate of treatment failure for buccal midazolam compared with rectal diazepam (30.3% vs 43%; p = 0.016)…studies reported respiratory depression with use of buccal midazolam in children…*”	American Epilepsy Society Guideline: Evidence-based guideline: treatment of convulsive status epilepticus in children and adults: report of the Guideline Committee of the American Epilepsy Society [67]
Musiime, 2004	Instrumental (frame a problem)	“*…In Uganda, E. coli, Salmonella and Shigella species caused most acute diarrhoea in children with high resistance to cotrimoxazole…*”	Integrated Management of Childhood Illness (IMCI): WHO recommendations on the management of diarrhea and pneumonia in HIV-infected infants and children [43]
Nabukeera, 2005	Instrumental (treatment option)	“*…data from five studies of children ages 2–18 years, conducted in Italy, Togo, Uganda and the United States, indicate a nearly 20% greater rate of adherence to ARV medication regimens among children whose HIV-positive status had been disclosed to them compared with children who were in treatment but had not been told their status…not significant, low quality, RR = 1.18, 95% CI .88–1.57)…*”	Guideline on HIV disclosure counseling for children up to 12 years of age [41]
Nakanjako, 2005	Instrumental (frame a problem and diagnostic option)	“*…A study in Uganda showed that, among adults who were offered HIV testing at a hospital (about half of whom were subsequently found to be HIV-positive), 83% were unaware of their HIV status, even though 88% had been to a health unit in the previous six months…*”	HIV/AIDS Programme: strengthening health services to fight HIV/AIDS: guidance on provider-initiated HIV testing and counseling in health facilities [68]
Njama, 2003	Instrumental (frame a problem)	“*…Prevalence among people from other malaria endemic areas is much lower… the risk of development of symptomatic malaria after screening is 20% to 40%...*”	Evidence-based clinical guidelines for immigrants and refugees [69]
Ojulong, 2007	Instrumental (frame a problem)	“*… Staphylococcus aureus resistance to methicillin* [in the] *African Region; Uganda 0% ... 54 tested isolates…*”	Anti-microbial resistance: global report on surveillance [70]
Orikiiriza, 2008	Instrumental (frame a problem)	“*…it is important to consider that some children with HIV infection who are started on antiretroviral therapy may experience a radiological deterioration despite clinical improvement due to immune reconstitution inflammatory syndrome (IRIS)…*”	Consensus statement on research definitions for drug resistant tuberculosis in children [71]
	Instrumental (diagnostic option)	“*…IRIS should be suspected in children with advanced immunosuppression who initiate cART and develop new symptoms shortly thereafter (within 3–6 months), despite evidence of good HIV control…in patients who have occult TB before cART initiation, TB may be unmasked by subsequent immune recovery…*”	Recommendations/guidelines for the prevention and treatment of opportunistic infections in HIV-exposed and HIV-infected children [62]
Wobudeya, 2008	Instrumental (frame a problem)	“*…Studies that suggest that breastfeeding can interfere with immune reaction to rotavirus vaccines. However, the results of these studies were not always statistically significant…*”	Recommendations for increasing the efficacy & coverage of the rotavirus vaccination program in Ghana [72]
Worodria, 2000	Instrumental (diagnostic option)	“*…a Ugandan study where patients suspected of PTB were found to have Pneumocystis jiroveci (38.6%), MTB (24%), pulmonary Kaposi’s sarcoma (11%), pyogenic bacteria (8%)…thus misdiagnosis of PTB can be reduced if appropriate investigations are conducted, particularly in countries with high HIV burdens…without a standardized clinical work up, rates of misdiagnosis have been estimated to be as high as 35% to 52%...*”	National guidelines not always followed when diagnosing smear-negative pulmonary tuberculosis in patients with HIV in Botswana [47]

Nearly all these cases of research use (91%) addressed at least one infectious disease (MDG 6), either HIV/AIDS, malaria, tuberculosis or diarrhoea, while two-thirds (63%) focused on child health (MDG 4). The types of studies that were cited in these documents were all clinical in nature, dominated by citations of observational studies (cross sectional, cohort, case control) that were about framing a health problem (68%). The only four randomised trials were cited seven times (32%) in policy documents that were about choosing a health intervention. None of the cited studies had a qualitative design component (Table 1).

### Qualitative synthesis of the documents and how research was used

#### Symbolic use of research

Our assessment of ‘symbolic use’ of research was based on the contextual analysis with positioning of the text in a manner suggestive of cementing a decision that has already been taken. In the first of the two instances, the British HIV/AIDS Association had already selected Amphotericin B as one of the many options for treating cryptococcal meningitis. The emergence of new evidence, including work from the dissertation of Kambugu 2002 [33], only served to reinforce this decision in the document entitled ‘British HIV Association opportunistic infection guidelines: in defence of amphotericin B deoxycholate’ [34], wherein they state that:

“*…We and others have previously demonstrated manageable and reversible renal impairment in cohorts of HIV-infected patients managed with AmBd at 0.7–1 mg/kg/day with careful pre-hydration and electrolyte monitoring and supplementation, without a requirement for renal replacement therapy…*”

In the second case of ‘symbolic use’, Marie Stopes International, a non-governmental organisation that promotes reproductive health, together with the School of Public Health of Columbia University, made a case for including misoprostol for management of abortion in the WHO Essential Medicines List. This Essential Medicines List applies to over 180 states worldwide which subscribe to WHO, particularly low-income countries where the subject of abortion is either illegal or abominable [35, 36]. In this context, the actors have an already formed an opinion of promoting safe abortion and apply this research, including work from the dissertation of Alia [37], to buttress their proposition. They state that:

“*…A wealth of scientific evidence…including numerous randomized and comparative clinical trials…supports our view that misoprostol should be included in the EML* [Essential Medicines List] *for this indication…for treatment of incomplete abortion and miscarriage…high success rates, in the range of 90 to 100%…*”

#### Instrumental use of research

Research was predominantly applied instrumentally, as depicted in Table 3. We highlight three instances where research was used instrumentally, namely to frame the problem by Musiime in 2004 [38], to assess a diagnostic option by Bakeera-Kitaka in 2002 [39], and to choose a treatment option by Mpimbaza in 2007 [40]. The work from Musiime was used in the ‘Integrated Management of Childhood Illness (IMCI): WHO recommendations on the management of diarrhea and pneumonia in HIV-infected infants and children’ [41]. Here, reference was made to frame the burden of diarrheal disease, in an internationally accepted guideline, as follows:

“*…In Uganda, E. coli, Salmonella and Shigella species caused most acute diarrhea in children with high resistance to cotrimoxazole…*”

In the second case of instrumental use, we observe that Bakeera-Kitaka’s thesis is cited as a diagnostic option in international guidelines for childhood pneumonia in persons living with HIV. This guide is entitled “The WHO Pocketbook of Hospital Care for Children: [Evidence behind the WHO Guidelines: hospital care for children: what is the etiology of pneumonia in HIV-infected children in developing countries?]” [42]. Two specific considerations assess the value of findings from this study, as follows:

Instance one: “*…a study of Ugandan children with severe pneumonia reported that those with PCP had a smaller head circumference compared to those without PCP (41.4 cm compared with 44.4 cm, p<0.0001)…*”

Instance two: “*…Similarly, Bakeera–Kitaka et al. found mean levels of 816 units/L in PCP compared with 568 units/L in others (p=0.004). Those died from PCP, in this study, had significantly higher levels than survivors. This is supported by evidence from developed countries…*”

The third case of instrumental use entailed the choice of a treatment option that was found to have more favourable outcomes for epilepsy (a neurological disease) in terms of effectiveness and safety. This work by Mpimbaza [40] was a randomised controlled trial and the largest study, providing for higher quality evidence. The specific phrases that cited these findings are captured below.

“*…the superior effectiveness and safety of buccal midazolam compared to rectal diazepam came from…three RCTs...the largest study of 330 children in Uganda found a lower rate of treatment failure for buccal midazolam compared with rectal diazepam (30.3% vs 43%; p = 0.016)…*”[43]

#### Emerging themes of how research was used

‘Synergistic relationships’ defined efforts by communities-of-practice aligning with government entities or other non-state actors to utilise research to proclaim a new consensus. Multiple cases of new guidelines for HIV diagnosis or treatment, or working papers for tuberculosis diagnosis or increasing access to safe abortion show two or more institutions advancing a common interest, and applying research in their quest. We observed a clear context of research use in synergies where two government entities teamed up with private communities of practice to apply evidence in issuing their new joint guidelines on paediatric HIV. The government entities were the United States National Institutes of Health (a research organisation) and the Centers for Disease Control and Prevention (a public health agency). The private organisations were the HIV Medicine Association of the Infectious Diseases Society of America, the Pediatric Infectious Diseases Society and the American Academy of Pediatrics.

Two pathways of research use illustrate ‘globalisation of local evidence’. In the longer loop, we observed locally generated evidence captured in global policy-related documents that are in turn exposed to the local Ugandan audience as guidelines for low- and middle-income countries. These policy-related documents were owned by international organisations such as WHO or Marie Stopes International and addressed global priority problems, HIV, tuberculosis, malaria and pneumonia, or access to safe abortion, all of which are relevant to Uganda. The second pathway terminates in the use of this locally generated research evidence solely in jurisdictions outside Uganda such as evidence-based clinical guidelines for immigrants and refugees by the Canadian Collaboration for Immigrant and Refugee Health, or HIV-related guidelines by the British HIV Association or Southern African HIV Clinicians Society.

‘Trade offs’ emerged in the use of research evidence. In a policy-related document entitled, ‘American Epilepsy Society Guideline: Treatment of Convulsive Status Epilepticus in Children and Adults’ [43], peer-reviewed research work from the dissertation by Mpimbaza [39] was among the largest of the five studies that reported benefits, but also contributed evidence towards harm of the intervention (midazolam), as illustrated below:

“*...The largest study of 330 children in Uganda found a lower rate of treatment failure for buccal midazolam compared with rectal diazepam, 30.3%* vs *43%; p = 0.016…two class III studies reported respiratory depression with use of buccal midazolam in children* [44, 45]*, in contrast to two class III studies, which reported no respiratory depression associated with use of buccal midazolam in the pediatric population* [40, 46]*…*” (pages 54 & 55).

A crucial observation was the use of ‘negative’ or ‘non-confirmatory’ results to inform new directions in the policy-related documents. In one case of research use, dissertation findings did not reach statistical significance but were pooled to increase power and precision of the effect estimate in the policy-related document. Results from the dissertation of Achan in 2007 [46], which compared the effect of intra-rectal to intravenous administered quinine, showed reduced mortality in Ugandan children, with a relative risk (RR) 0.77 and 95% confidence interval of (CI) 0.22–2.72. Achan’s work was included in the document ‘Treatment of African children with severe malaria: towards evidence-informed clinical practice using GRADE’. This study contributed 42.7% of the data to the meta-analysis results (RR 0.55, 95% CI 0.24–1.24) that had a narrower CI. In a second case of research use that applied ‘negative results’, work from the dissertation of Wobudeya in 2008 [44] was included in a working paper, the ‘Recommendations for Increasing the Efficacy & Coverage of the Rotavirus Vaccination Program in Ghana’ [47]. Wobudeya’s study contributed to the body of evidence that generated a hypothesis for further testing.

“*…Studies suggest that breastfeeding can interfere with immune reaction to rotavirus vaccines. However, the results of these studies were not always statistically significant…a priority for clinical investigators should be to conduct trials that examine whether withholding breastfeeding 30 minutes before and 30 minutes after immunization has an effect on immune response to the vaccine…*” ([47] p. 5, 6).

## Discussion

### The principal findings

The key message from our multiple case study is that research products from dissertations of post-graduate students at MakCHS are used in EIHP. Secondly, our findings portray how results were used either instrumentally to choose treatment or diagnostic options, or even to defend these choices (symbolically) in order to address the priority health problems in line with the MDGs. Third, we identified emerging themes from the content, including ‘synergistic relationships’ among organisations or interest groups using these research products, among many, to advance a new consensus; ‘globalisation of local evidence’ as a pathway of the research products; ‘trade-offs’ in the application of the benefits and harms from the research results; and the importance of ‘negative results’ in strengthening the overall body of available evidence or depicting information gaps or generating new hypotheses.

### Findings in relation to other studies

This study mapped the use of research from post-graduate students’ dissertations in EIHP and described how this research was used go beyond what is known so far. Most studies about post-graduate students’ dissertations have focused on publication outputs, strategies to increase publication and contextual factors that influence this process [11–14]. To this end, our finding of very few cases of research use is corroborated by existing evidence on students’ research that underscores a suboptimal publication rate, which makes research less accessible for use, across different contexts, but more so in lower income countries [48–50]. While previous work specific on ‘research use’ did not discriminate on whether students’ research was used, their methods laid the foundation for our study [3, 16, 51]. Work in Canada, Cameroon and Uganda generally showed that research is indeed used, mainly instrumentally, and further depicted a general increase in citation of research after the global initiatives to promote EIHP in Mexico and Mali [6]. An important observation is that there was hardly any use of evidence syntheses (e.g. systematic reviews or evidence briefs for policy) and similar findings have been reported before even at WHO [45]. Rather, we observed that students’ research contributed to the body of evidence that was used in policy-related documents, including in systematic reviews that underscored their unique contribution.

### Strengths and limitations

To the best of our knowledge, this is the first study that has attempted to unearth the direct linkage of students’ research to the public health policy process. Further, using a multiple case study approach, we have employed robust methods combining policy sciences theoretical frameworks, quantitative analysis and qualitative content analysis techniques to decipher the phenomenon of research use in the context of post-graduate students research [29]. However, we found only cases of research use in international domains and acknowledge the limitations of our methods in capturing ‘local use’ in Uganda, possibly a function of poor documentation or archival of policy-related documents in the world-wide-web or the lack of explicit citations of research in such documents. Not least, we did not characterise ‘symbolic use’ in its strictest sense and, broadly, our methodology was not suitable to assess conceptual use, neither was conceptual use captured. It would not be expected that all instrumental use of research would be explicitly documented, as policy documents often do not contain references to the research that underpins them. Further, it is less likely that conceptual research use would be visible, or that symbolic use would be overt. As such, it is likely that the results underestimate the use of these dissertations in informing policy. Indeed, there are variants in the models of research utilisation for which we did not exhaust our analysis as we observed that, generally, these models manifest differences in areas of emphasis, but define a complex and lengthy process that requires innovative and dedicated action on the part of knowledgeable strategic planners and change agents. We identify this as an area that could be pursued in future studies. Finally, we take cognisance of the shift from MDGs to Sustainable Development Goals in 2016 [52], during the course of this study. The phrase ‘unfinished MDG business’ was coined to reflect the failure of some countries to meet their 2015 targets. Given that Uganda did not achieve its targets, particularly with regards to maternal and child health, we are thus satisfied that these study findings remain applicable in this context.

### Implications for policy

The low uptake of students’ research into policy may be explained by a misperception of lower quality data or results which may not be exciting to the users [53], or by the absence of immediate returns for students who take research dissertations as rituals to accomplish degree training and immediately embark on job hunting [48]. However, in a systematic review of meta-analyses comparing unpublished and published data, there was no evidence that the quality of research in unpublished dissertations was inferior to that in journal articles [54, 55]. Our findings show that even ‘negative or non-confirmatory results’ from students’ dissertations augment weak findings at the global level by improving certainty of the estimate or generating new ideas for further research. Scholarship funders and universities could consider placing more value in efforts to promote research use for the betterment of society. Engaging graduate students in priority-setting exercises, deliberative workshops and generation of evidence syntheses as a requirement for obtaining their degree could be explored as sound research investment models [56] beyond publication outputs [14].

### Implications for future research

While previous research emphasised publication productivity of students’ dissertations [11–14, 48–50], our work has advanced this field towards ‘research use’. We have not only analysed the ‘citometrics’ but also enlightened how research was used as well as the pathways and contexts of use of students’ research. Future studies could examine local relevance and how students’ research informs decision-making in technical working groups at Ministries of Health, relevant committees in Parliament or Local Governments. A post-graduate tracking study to find how they applied their dissertation results in their work environments in terms of influencing policy or embedding students research in the local existing structures such as Ministry of Health technical working groups would be informative. This will entail the use of broader qualitative methods such as key informant or in-depth interviews, in addition to documentary analysis of health-related national strategic plans, development plans, performance reports and proceedings of decision-making meetings. Further work could assess the extensiveness of Weiss’s framework including symbolic or political as well as conceptual use of research.

Indeed, a lot of changes have occurred at MakCHS as well as in the knowledge translation environment in Uganda in the recent past [57]. It might be instructive to conduct a similar study that looks at the subsequent period from 2010 onwards, with particular focus on doctoral students’ research.

## Conclusions

There is evidence that research from dissertations of post-graduate students at MakCHS is used in EIHP to a limited extent. These results probably underestimate the use of this research, as they do not include the forms of research use that are not documented or are more difficult to identify. Future studies could employ our robust approaches to study the loop of local use of students’ research from MakCHS, other universities in Uganda and the region, as well as consider doctoral students’ research.

## Abbreviations

EIHP: Evidence-informed health policy
MakCHS: Makerere University College of Health Sciences
MDG: Millennium Development Goals
